# EVALUATION OF THE ILLINOIS PUBLIC HEALTH ASSOCIATION ALZHEIMER’S DISEASE EARLY DETECTION AND AWARENESS CAMPAIGN

**DOI:** 10.1093/geroni/igad104.3312

**Published:** 2023-12-21

**Authors:** Julie Bobitt, Zohaib Zahir, Krissy Roseberry

**Affiliations:** University of Illinois Chicago, Chicago, Illinois, United States; Illinois Public Health Association, Springfield, Illinois, United States; Illinois Public Health Association, Springfield, Illinois, United States

## Abstract

Of the 55.8 million adults aged 65 and over in the US, nearly 12% are at risk for or have subjective cognitive decline (SCD), yet more than half have not seen a healthcare provider about their SCD. Furthermore, studies show that fewer than 50% of individuals with Alzheimer’s or a related dementia (ADRD) have been diagnosed by a physician. Missed diagnoses of dementia are more common among Blacks/African Americans than Whites and both Black/African American and Latinx individuals with cognitive impairment are less likely than Whites to report that a physician has diagnosed them. To adequately address community needs for ADRD early detection and awareness, the Illinois Public Health Association (IPHA) established the Alzheimer’s Disease Early Detection and Awareness Campaign and partnered with community-based organizations (CBOs) and their community health workers to educate underserved populations about the risk for ADRD and the importance of early screening, and to assist clients in achieving referral to ADRD care services. To evaluate the campaign, IPHA partnered with the University of Illinois Chicago. We used the RE-AIM (Reach, Effectiveness, Adoption, Implementation, Maintenance) implementation outcomes framework to guide the evaluation and share quantitative and qualitative results including the number of participants that reached our target audiences, effectiveness of the program on participant knowledge and behaviors, adoption of the campaign elements by the CBOs, implementation barriers and facilitators, and intentions for program maintenance. Results and lessons learned can be used to inform awareness and education efforts in other communities with special emphasis on reaching underserved populations.

